# Single-Cell Kinetic Modeling of β-Lapachone Metabolism in Head and Neck Squamous Cell Carcinoma

**DOI:** 10.3390/antiox12030741

**Published:** 2023-03-17

**Authors:** Andrew D. Raddatz, Cristina M. Furdui, Erik A. Bey, Melissa L. Kemp

**Affiliations:** 1 Wallace H. Coulter Department of Biomedical Engineering, Georgia Institute of Technology, Emory University, Atlanta, GA 30332, USA; 2 Department of Internal Medicine, Section on Molecular Medicine, Wake Forest School of Medicine, Winston-Salem, NC 27101, USA; 3 Wood Hudson Cancer Research Laboratory, Newport, KY 41071, USA

**Keywords:** head and neck squamous cell carcinoma, scRNA-seq, redox biology, ROS, systems modeling, β-lapachone

## Abstract

Head and neck squamous cell carcinoma (HNSCC) cells are highly heterogeneous in their metabolism and typically experience elevated reactive oxygen species (ROS) levels such as superoxide and hydrogen peroxide (H_2_O_2_) in the tumor microenvironment. Tumor cells survive under these chronic oxidative conditions by upregulating antioxidant systems. To investigate the heterogeneity of cellular responses to chemotherapeutic H_2_O_2_ generation in tumor and healthy tissue, we leveraged single-cell RNA-sequencing (scRNA-seq) data to perform redox systems-level simulations of quinone-cycling β-lapachone treatment as a source of NQO1-dependent rapid superoxide and hydrogen peroxide (H_2_O_2_) production. Transcriptomic data from 10 HNSCC patient tumors was used to populate over 4000 single-cell antioxidant enzymatic network models of drug metabolism. The simulations reflected significant systems-level differences between the redox states of healthy and cancer cells, demonstrating in some patient samples a targetable cancer cell population or in others statistically indistinguishable effects between non-malignant and malignant cells. Subsequent multivariate analyses between healthy and malignant cellular models pointed to distinct contributors of redox responses between these phenotypes. This model framework provides a mechanistic basis for explaining mixed outcomes of NAD(P)H:quinone oxidoreductase 1 (NQO1)-bioactivatable therapeutics despite the tumor specificity of these drugs as defined by NQO1/catalase expression and highlights the role of alternate antioxidant components in dictating drug-induced oxidative stress.

## 1. Introduction

Head and neck squamous cell carcinoma (HNSCC) is one of the most prevalent types of cancer globally [1]. Prophylactic measures such as HPV vaccination and the reduction of alcohol consumption and smoking are improving outcomes; however, five-year survival rates of HPV-negative HNSCC remain lower than 60% [2]. While the etiology of HNSCC and anatomical locations within the oral cavity epithelial tissue are diverse, a hallmark of this cancer is elevated oxidative stress [3]. Hydrogen peroxide (H_2_O_2_) at physiological concentrations is important as a second messenger for many signaling processes, including the MAPK, PI3K, NF-κB, and HIF pathways [4,5,6,7,8]; however, H_2_O_2_ at higher levels promote tumorigenesis by causing genomic instability and proliferative signaling [9]. If H_2_O_2_ levels are elevated even further, the level of oxidative stress cannot be managed and cells go through one of several cell death mechanisms, including necrosis, apoptosis, and ferroptosis [10,11]. Cancer cells manage levels of H_2_O_2_ through multiple antioxidant enzyme systems [12], and under sustained oxidative stress will transcriptionally upregulate several antioxidant enzymes via the Keap1-Nrf2 axis [13,14]. One treatment strategy is to selectively target cancer cells through the generation of reactive oxygen species (ROS) and disrupt the delicate balance these cells have between their higher antioxidant capacity and higher oxidant levels [15,16,17,18,19]. A unique approach to this strategy is utilizing enzyme-activatable quinone drugs to generate superoxide and H_2_O_2_. NAD(P)H:quinone oxidoreductase 1 (NQO1) is an enzyme canonically involved in detoxifying reactive quinones by reducing them to their hydroquinone form; NQO1-activatable quinone drugs are oxidized back to their quinone form following NQO1 reduction, leading to a cycling of the drug and production of superoxide radicals. Because NQO1 is a quinone-reducing enzyme that is upregulated by Nrf2 [20], the rationale is that this approach should selectively target cancer cells that have constitutive Nrf2 activation. Furthermore, it can be argued that the generation of acute superoxide and H_2_O_2_ by NQO1-activatible therapeutics can cause a positive feedback response leading to more NQO1 expression and enhanced lethality of these compounds, because high ROS levels activate nuclear translocation of Nrf2 [21]. Numerous studies have shown the benefit of these types of drugs alone, and targeting additional antioxidant and survival systems concurrently can improve the efficacy of the drug [22,23,24,25]; however, there is debate as to whether the currently considered metric of NQO1:catalase expression or activity ratio is useful for identifying tumors susceptible to NQO1-activatable quinone drugs [26,27,28,29]. To improve our understanding of the complex interplay between various antioxidant systems and the production of ROS by NQO1-activatable drugs, we developed and analyzed a differential equation model based on enzyme kinetic mechanisms that leverages the diversity of single-cell expression levels relevant to cancer redox systems. Furthermore, we explored potential uses for such a model by initializing parameter and species values using scRNA-seq data as a way to understand intratumor and patient variability in response to this type of chemotherapeutic intervention.

## 2. Materials and Methods

### 2.1. Ordinary Differential Equation Model Construction

The redox system ODE model was built upon a previously published model originally developed to describe H_2_O_2_ clearance within Jurkat T cells in response to a bolus of extracellular H_2_O_2_ addition [12]. MATLAB R2020b was used, and the ode system was solved with the ode15s solver with a max step of 1 s and an absolute tolerance of 10^−8^. The additional species included in new reactions were: oxidized extracellular β-lapachone (β-lap^ext^), intracellular O_2_^•−^, oxidized intracellular β-lapachone (β-lapQ), reduced intracellular β-lapachone (β-lapHQ), semioxidized intracellular β-lapachone (β-lapSQ), and glutathionylated intracellular β-lapachone (β-lap-GSH). New reaction rate terms are provided in Table 1. Supplemental Tables S1 and S2 list the complete parameters and initial values, respectively, used within the ODE system that were updated from the model originally characterized for Jurkat cells [12].

### 2.2. Sensitivity Analysis

Sensitivity values were calculated by increasing or decreasing parameter values by 10%, running the ODE solver for a simulated 2 h, and using the following formula where S_i,j_ represents the sensitivity of variable x_i_ to parameter k_j_. If S_i,j_ is negative, it represents x_i_ changing in the opposite direction of k_j_.

(1) $$S_{i,j}\left(t\right)=\frac{\partial x_{i}\left(t\right)}{\partial k_{j}}$$

### 2.3. Single-Cell RNA Sequencing Data Analysis

HNSCC scRNA-seq data were collected from the gene expression omnibus (GEO Accession: GSE103322). This data had already been preprocessed to exclude cells with fewer than 2000 genes detected or an average expression level below 2.5 of a curated list of housekeeping genes [30,31]. The data set had also been clustered into malignant and non-malignant cells based on their expression of epithelial markers. Of the data from 18 patients, we retained the 10 patients’ transcriptomes that contained the most cells classified as malignant cells as previously performed [30,32]. t-SNE dimensional reduction was performed using the scikit-learn python library *scikit-learn.TSNE* with 2 components and PCA initialization. Enzyme abundance calculations from scRNA-seq data were performed as previously described [33]. Briefly, kinetic rate constants from a mechanistic model of RNA production, RNA degradation, protein production, and protein degradation were used to determine equilibrium protein abundances given RNA levels. Thirteen of the 14 protein values were derived using this mechanistic model and using the data in the original paper; these 13 predicted protein copy numbers correlated with the actual protein copy numbers with an R^2^ of 0.933. For AQP3, for which these rate constants were not given, linear regression between RNA and protein was used to estimate protein abundance. Partial least squares regression (PLSR) was performed with log-transformed and zero-mean unit variance standardized data in SIMCA 17 (Sartorius). Plots were generated using Seaborn and Matplotlib python libraries. The kernel density estimate plot was generated with default parameters using *seaborn.kdeplot*. Scipy was used to conduct the Welch’s *t* tests with *stats.ttest_ind* and equal_variance set to False.

## 3. Results

### 3.1. A Systems-Level Model of Superoxide and H_2_O_2_ Generation by Quinone Cycling

We developed our model system to encompass three main aspects:

(1) sets of critical H_2_O_2_-stabilizing antioxidant systems;
(2) metabolism of the xenobiotic drug β-lapachone;
(3) the permeation of key species across membranes of the cell, including organelle-specific transport.

**Figure 1:**
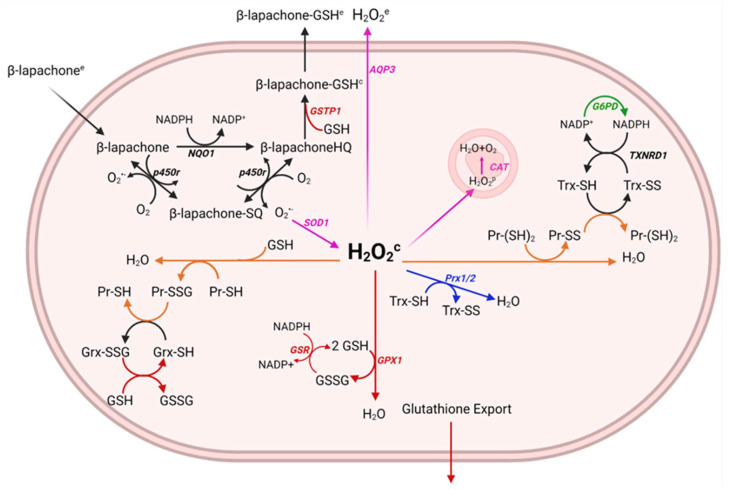
Generation of a relevant model of drug metabolism and hydrogen peroxide clearance pathways. The metabolism of β-lapachone by NQO1 results in the generation of superoxide (O_2_^•−^) and the oxidation of NADPH. Superoxide dismutase 1 (SOD1) in the cytosol converts the superoxide to hydrogen peroxide (H_2_O_2_) which is converted to water and oxygen by antioxidant systems including the peroxiredoxin/thioredoxin/thioredoxin reductase/sulfiredoxin system, the glutathione peroxidase/glutathione/glutathione reductase system, catalase, and the oxidation of free protein thiols. NADPH often serves as the reductant for cycling these antioxidant enzymes and is used to reduce β-lapachone, thus, canonical metabolic reactions involved in the production of NADPH are also included, such as glucose−6-phosphate-dehydrogenase (G6PD). Intermediate protein oxidation steps not shown in the diagram.

We assumed that mitochondrial H_2_O_2_ production would remain constant due to basal respiratory metabolism, and mitochondrial antioxidant systems were not included, nor did we factor in activation of NADPH oxidases as a source of superoxide as it has been shown that β-lapachone treatment does not alter NADPH oxidase activity leading to superoxide levels from NOXs remaining similar with or without treatment [34]. Another assumption made was that due to high catalytic rates of NQO1 and antioxidant enzymes, 2 h of simulated time was sufficient to capture the dynamics of the system. The relatively short period of simulated time allowed us to ignore transcriptional and translational regulation, such as how increased cellular oxidation would trigger Nrf2 nuclear translocation and upregulation of antioxidant genes including NQO1; therefore, total enzyme concentrations were assumed constant. The system and directionality of reactions and transport are shown in Figure 1.

### 3.2. Head and Neck Squamous Cell Carcinoma Cells Exhibit Heterogeneity of Redox Gene Expression

**Figure 2:**
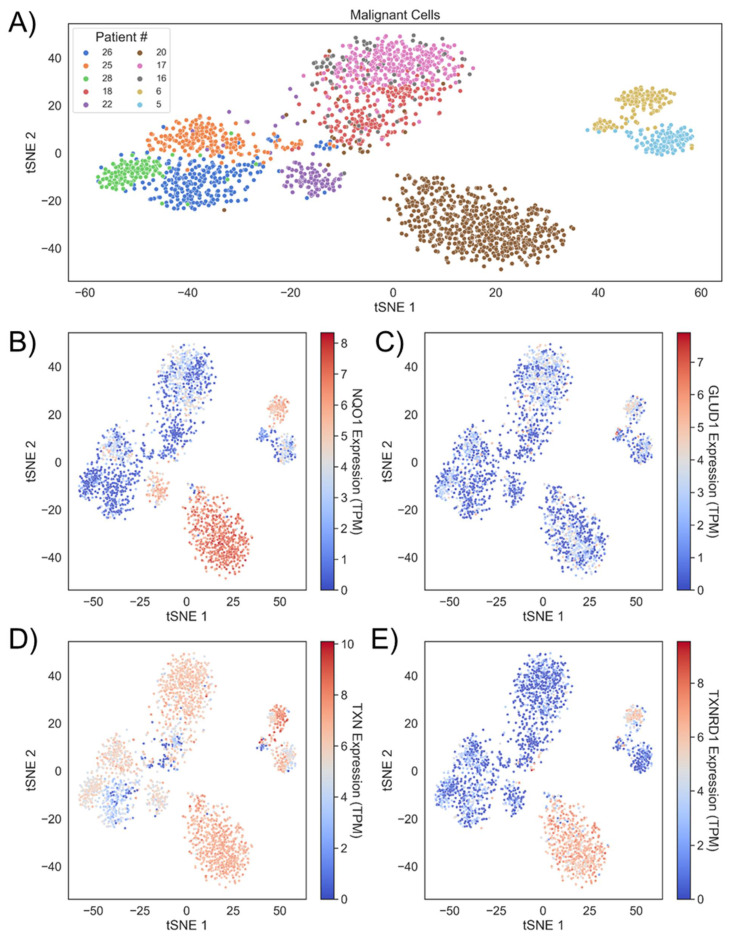
Head and neck cancers demonstrate intratumor and intertumor redox heterogeneity. (**A**) Malignant cells from 10 HNSCC patients cluster together based on redox profiles. Patients are identified by the numbering used in the source data (GEO Accession: GSE103322) (**B**) Clusters colored by NQO1, (**C**) GLUD1, (**D**) TXN, and (**E**) TXNRD1 expression.

We sought to understand how variation in redox profiles of in vivo HNSCC tumors may reflect the distributed control of H_2_O_2_ clearance in tumor cells. To take advantage of new highly resolved omics technologies that provide rich tumor characterization, we analyzed scRNA-seq data from 10 HNSCC patients originally collected by Puram et al. [30]. In this dataset, there is a varying degree of cell type representation from each patient, likely due to both cross-patient tumor microenvironment heterogeneity and preprocessing of scRNA-seq reads for quality control. After splitting the dataset into malignant and non-malignant cells and reducing the variables to just 35 redox genes represented in our quinone cycling systems model, t-SNE clustering revealed that malignant cells tended to cluster by patient (Figure 2A), suggesting that there were distinct, patient-based tumor redox profiles. After clustering and shading samples by gene expression, we observed that while interpatient heterogeneity was likely the cause for patient-specific clustering, intrapatient heterogeneity still existed and could be a source of why cells within a tumor can have different drug responses. This heterogeneity was observed when inspecting NQO1, GLUD1, TXN, and TXNRD1 expression (Figure 2B–E). GLUD1 was included due to its ability to generate high production rates of NADPH as demonstrated by flux balance analysis studies [35]. With this knowledge of heterogeneity between and within patient tumors, we leveraged redox transcriptional profiles per cell per patient to explore potential H_2_O_2_ buildup on cell- and tumor-based scales.

### 3.3. Initializing Single-Cell ODE Models with scRNA-Seq

While scRNA-seq data has been widely used for exploratory data analysis and to understand gene expression correlations within developing tissues and cancer, this form of characterization has only recently been used to inform mechanistic kinetic models [36]. We generated unique cell-based ODE systems using the previously analyzed scRNA-seq data to populate enzyme abundances that dictate kinetic reaction rates. With the redox transcriptional profiles of almost 5000 cells from 10 patients, we first estimated the redox protein profiles as previously described [33,37] and imported these protein concentrations and related rate constants into our ODE model followed by simulation of the redox metabolism for each cell undergoing acute H_2_O_2_ generation by β-lapachone treatment. Specifically, AQP3, GSR, TXNRD1, NQO1, SOD1, POR, G6PD, and GLUD1 expression levels were used to adjust reaction rate constants by multiplying the rate constants by the percent change in the single-cell expression from the average. G6PD and GLUD1 both generate NADPH and were combined into a single reaction in the model, and the impact of their expression levels on the kinetic rate constant was additive. GPX1, CAT, PRX1, PRX2, TXN, and GLRX expression levels were used to estimate initial enzyme abundances. PRX1 and PRX2 expression levels were combined and represented a single reaction in the model, and the impact of their expression levels on the initial enzyme abundances was additive. All other parameters and species levels were kept from prior modeling [12].

### 3.4. Sensitivity Analysis Shows H_2_O_2_ Production Is Insensitive to Individual Enzymatic Parameters

**Figure 3:**
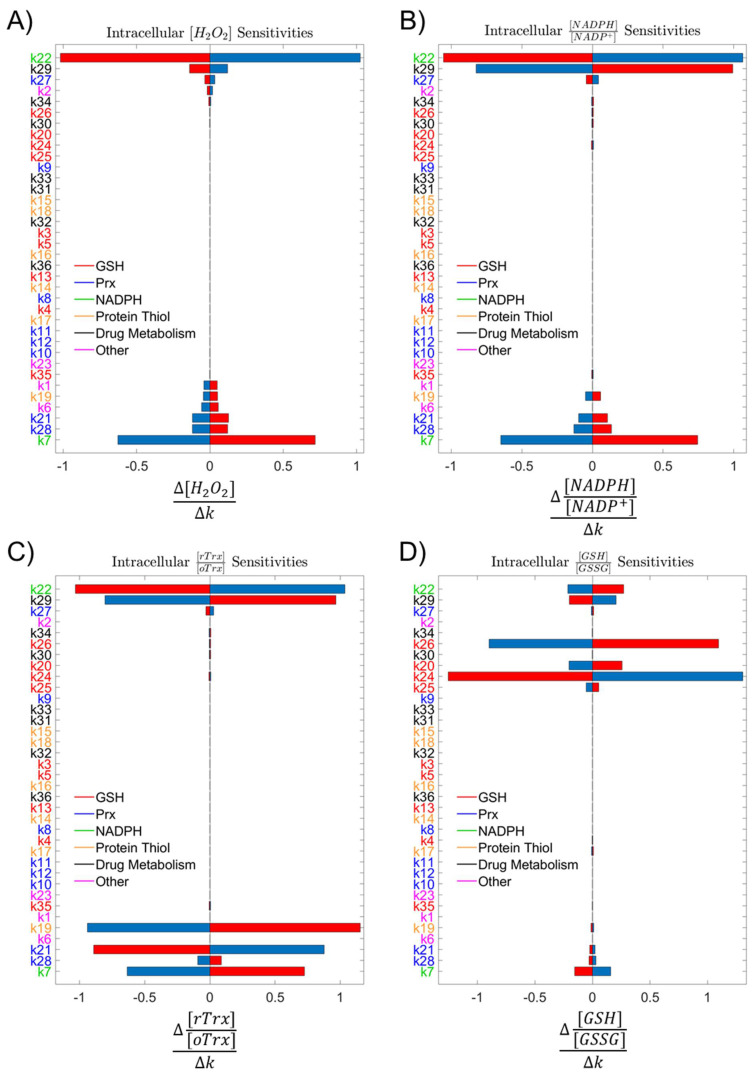
Sensitivity analyses. (**A**) Analysis of system sensitivity to single-parameter 10% perturbations colored by the antioxidant subsystem showed low sensitivity of (**A**) intracellular H_2_O_2_, (**B**) NADPH:NADP^+^, (**C**) Trx-SH:Trx-SS, and (**D**) GSH:GSSG at 2 h to any single parameter. Blue bars represent an increase in the parameter by 10%, and red bars represent a decrease in the parameter by 10%.

After constructing the ODE system, we sought to understand how influential each simulation parameter was on our system by performing a sensitivity analysis. We assessed the effect on intracellular H_2_O_2_ as the output variable of interest by altering model parameters up or down 10%. With most sensitivities remaining below 1 and H_2_O_2_ only being somewhat sensitive to several parameters, we concluded that no single parameter could alter H_2_O_2_ production significantly (Figure 3A).

Parameter labels colored by the antioxidant subsystem also indicated that no single antioxidant system was controlling a majority of the H_2_O_2_ scavenging load. Expanding the number of outcomes to include redox ratios of NADPH to NADP^+^, reduced thioredoxin to oxidized thioredoxin, and reduced glutathione to oxidized glutathione allowed us to assess the impact of these parameters on alternative indicators of redox status within the cell. The distribution of parameter importance in the sensitivity analyses across multiple redox mechanisms suggested that the reductive capacity of a cell was robust, and no single antioxidant enzyme system was predominantly responsible for clearance of H_2_O_2_ (Figure 3B–D).

### 3.5. Comparison of H_2_O_2_ Accumulation in Healthy and Cancer Cells Identifies Patients with Greatest Potential for Targeted Therapy

**Figure 4:**
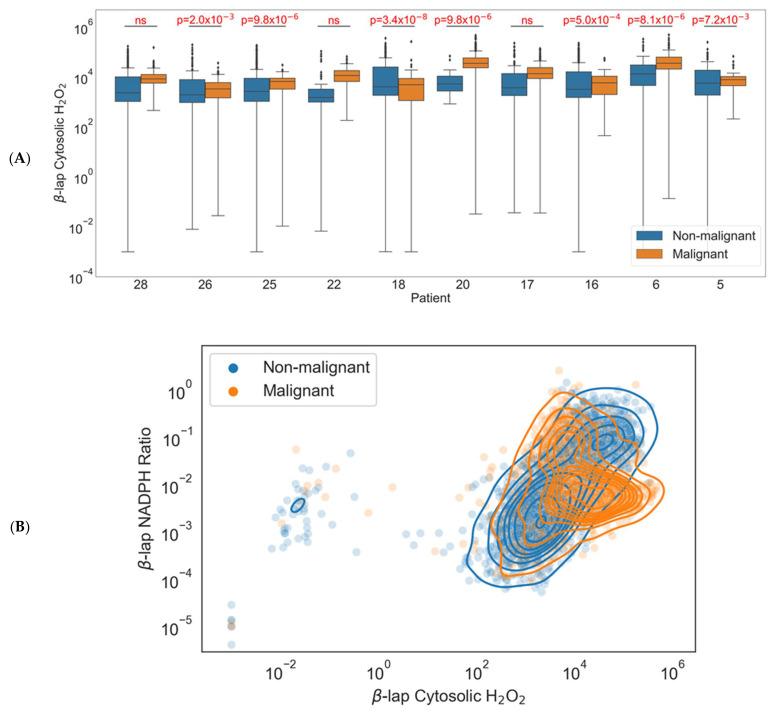
**Model results using single-cell gene expression values.** (**A**) Differences between cytosolic H_2_O_2_ in healthy and malignant cells under β-lapachone by patient. Boxes are defined by the interquartile range (IQR) with the line inside representing the mean. Upper whiskers are defined by adding 1.5 times the IQR to the third quartile, and lower whiskers are defined by subtracting 1.5 times the IQR from the first quartile. Black diamonds represent outlier samples outside the whiskers. (**B**) Differences between the NADPH:NADP^+^ ratio and cytosolic H_2_O_2_ in healthy and malignant cells under β-lapachone.

Using this new system of generating single-cell ODE models, the redox profiles of individual cells within HNSCC can vary greatly and result in a range of H_2_O_2_ spanning many orders of magnitude. After removing simulations that were unstable, we had 4243 single-cell simulation outputs across all ten patients. All ten patients showed a trend of more H_2_O_2_ generated by the malignant cells relative to that of the normal cells, with seven patients exhibiting statistically significant differences as measured by two-tailed Welch’s *t* test (Figure 4A).

Additionally, when comparing both H_2_O_2_ output and endpoint NADPH:NADP^+^ ratios across the 4243 cellular models, we generally saw higher H_2_O_2_ levels in cancer cells but no clear trend in NADPH:NADP^+^ ratios (Figure 4B). The denser malignant population may indicate tighter redox regulatory control amongst the malignant cells. Alternatively, the less dense non-malignant population may simply reflect the fact that it includes all other cell types within the tumor (e.g., tumor-infiltrating T lymphocytes, macrophages, stromal cells). This shift demonstrates a potential for using single-cell profiling to select patients for treatment with this targeted chemotherapy based on their redox profile. For the three patients where treatment induced H_2_O_2_ in both healthy and malignant cells without a statistically significant difference, the therapy may induce normal-tissue toxicity, impacting treatment and long-term quality of life.

### 3.6. Initializing Single-Cell ODE Models with Patient HNSCC Scrna-Seq Identifies Proteins Correlated with NADPH Ratio

**Figure 5:**
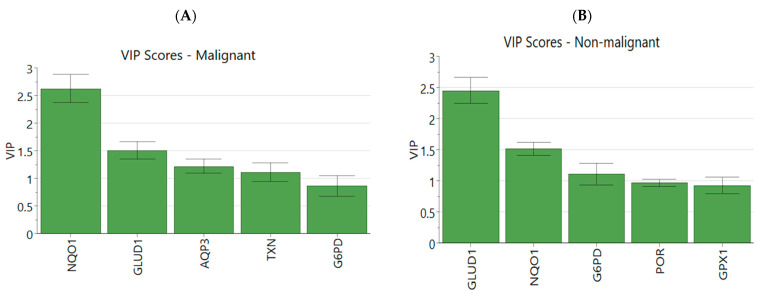

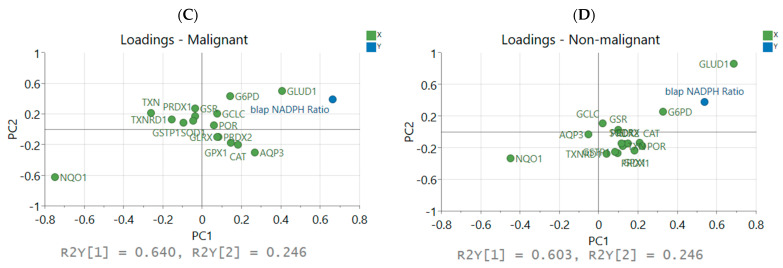
**Partial least squares regression VIP scores and loadings.** (**A**) Genes with top 5 VIP scores in the PLSR model in malignant simulations and (**B**) non-malignant simulations. (**C**) Breakdown of output into individual variables and loadings for each X and Y variable in malignant PLSR model and (**D**) non-malignant PLSR model.

Cytosolic NADPH redox ratio after a 2 h simulation was used as the dependent Y-variable in partial least squares regression to probe the correlations between the protein concentrations within the model and the output variable. With four and three components, respectively, both the malignant and non-malignant regression models were able to achieve both high explained output variance (non-malignant R^2^Y = 0.891, malignant R^2^Y = 0.871) and goodness of prediction (non-malignant Q^2^ = 0.886, malignant Q^2^ = 0.858). VIP scores identified NQO1, GLUD1, AQP3, TXN, and G6PD as the most important variables in the malignant model (Figure 5A) and GLUD1, NQO1, G6PD, POR, and GPX1 as the most important variables in the non-malignant model (Figure 5B). An interesting result was that GLUD1 had a higher VIP score in the non-malignant cells than NQO1 did, indicating that the outcome of drug treatment in non-malignant cells was more dependent on the production capacity of NADPH than the expression of the NADPH-reducing enzyme involved in drug metabolism.

Collectively, the distribution of redox enzymes across principal components 1 and 2 differed between the two statistical models (Figure 5C,D) but there were several similarities. The distribution of NQO1 and CAT loadings in latent space reflected prior reports of NQO1/CAT not correlating well with LD_50_ values of *β*-lapachone across a diverse HNSCC panel [22]. Additionally, the importance of AQP3 in the malignant model demonstrated the impact that diffusion of H_2_O_2_ across the cell membrane can have on the oxidative stress within a cell. Anticorrelation with the thioredoxin/peroxiredoxin component to H2O2 clearance was reflected by negative PC 1 scores in the malignant model. NADPH-producing enzymes GLUD1 and G6PD and the main drug-metabolizing enzyme NQO1 were most correlated with NADPH ratios, and most other antioxidant enzyme expression levels were less important due to low magnitude of their loading weights, i.e., proximity to the origin (Figure 5C,D).

## 4. Discussion

Because the main mechanism of action by NQO1-activatable drugs is the generation of superoxide and H_2_O_2_, the ability for a cancer cell to manage these oxidants is a critical metric for chemotherapeutic response. The NQO1:CAT ratio has been proposed as a predictive variable of NQO1-activatable drug success, but the utility of this metric is debated. Bey et al. in 2013 first suggested that NQO1:CAT could be useful after finding that the use of exogenous catalase reduced the effects of β-lapachone in breast cancer [26], and higher NQO1:CAT was observed in NSCLC tumors that responded to treatment than in matched healthy tissue [27]. In 2017, it was reported that the LD_50_ of β-lapachone did not correlate with NQO1:CAT in head and neck cancer [22]. Additionally, while NQO1:CAT was not directly measured, inhibition of catalase and GSH did not lead to sensitization of KEAP1-mutated NSCLC during β-lapachone treatment, while inhibition of TXNRD and SOD1 sensitized cancers [29]. A recent TCGA analysis revealed higher NQO1:CAT levels in hepatocellular carcinoma (HCC) than in matched healthy tissue, and the authors reported that the high-NQO1 patient cohort had lower survival [38]. These studies serve to highlight the complexity of the antioxidant system in the context of NQO1-activatable drugs like β-lapachone and suggest that the current approach for identifying how well a solid tumor would respond to the treatment is underdeveloped. In this report, we generated a more accurate model of superoxide and H_2_O_2_ generation and scavenging under β-lapachone conditions by including additional antioxidant systems in an ODE-based approach in which H_2_O_2_ generation was a surrogate for drug potency. Including additional antioxidant systems and the kinetic information of enzymes simultaneously allowed us to predict measures other than NQO1:CAT that could serve as an indication of β-lapachone success.

When building a model to represent a biological system, there are always simplifications and assumptions that must be made using field expertise. Transcriptional regulation of the Keap1–Nrf2 axis on the scale of hours to days was not accounted for, in which the positive feedback of H_2_O_2_ activation of Nrf2-targeted genes resulted in enhanced NQO1 expression [14,39]. Another major assumption used was that mitochondrial antioxidant systems would not reduce the large amount of ROS in this chemotherapeutic context due to the cytosolic location of NQO1 [40]. Work done by Ma et al. shows that mitochondrial-targeted β-lapachone produces mitochondrial ROS using MitoSOX, while 3-hydroxy β-lapachone, which is not mitochondrially targeted, produces no substantial mitochondrial ROS [41]. This allowed us to omit antioxidant enzymes expressed in the mitochondria such as SOD2, PRDX3, PRDX5. We did, however, find relatively high sensitivities of H_2_O_2_ permeabilities in the model, indicating the importance of how quickly a cell can export ROS during treatment. While H_2_O_2_ can passively diffuse through the phospholipid bilayer, it is also known to utilize aquaporin membrane proteins to travel through the plasma membrane [42,43,44,45]. Because of the high sensitivities, measuring aquaporin expression levels could serve as a useful indicator of β-lapachone success.

When generating enzymatic models, direct expression levels of proteins can be acquired experimentally or from published datasets of other scientists’ experiments. We chose an alternative strategy by estimating protein abundance based on scRNA-seq mRNA levels. Because transcriptional levels do not directly correlate to protein levels, we used a quantitative pipeline to estimate protein abundances that leverages previously published data from Schwanhausser et al. [33,37]. This allowed us to automate the generation of an ODE system specific to each cell sequenced in the scRNA-seq data. From our initial exploration of the scRNA-seq data, we observed that the cells clustered by patient regardless of if they were healthy or cancerous, similar to the results of an analysis conducted by Xiao et al. [32], so we concluded that each tumor was composed of a population of cells that were similar in redox profile. However, when analyzing the expression of each antioxidant enzyme within these clusters, the overall antioxidant capacity or diversity of each tumor was unclear due to varied levels of each antioxidant enzyme. Our ODE model was able to stratify the patient tumors based on the differences in the expected response of healthy and cancerous cells to β-lapachone, shedding some light on the complex nature of redox systems. Because we used scRNA-seq data that had transcriptomes of both non-malignant and malignant cells, we were able to assess the relative dependence of these two cell populations on their antioxidant enzyme expression under oxidative stress. Upholding the current paradigm that cancers experience higher levels of oxidative stress, we did observe higher average levels of H_2_O_2_ within the cancer cell models on average compared to those of the non-malignant cell populations within a given patient tumor. Additionally, the fact that some patients had significant differences between these healthy and malignant cell populations while others did not reinforces the notion that this drug is optimally used in a personalized manner for those patients that would reflect selective targeting to malignant tissue. When the contours of the two cell populations were plotted in a 2D phase space of the two output variables, cytosolic H_2_O_2_ and NADPH ratio, we found that they overlapped quite closely, but the cancer cell range was more compact. Non-malignant cells represent a repertoire of components found in the tumor microenvironment ranging from fibroblasts to macrophages, and thus a diversity of responses to an oxidative insult is expected. In contrast, the cancer cell phenotype could serve as a survival advantage of the cancer cells in oxidative environments. Similarly, while our comparisons of non-malignant and malignant cells’ redox state after simulated treatment aggregated the cell population per patient, we observed wide variability within each group. Some non-malignant cells showed a more oxidatively stressed state than malignant cells in the same tumor did. While cancer cells are typically seen as being more oxidized, these results predict that tumor heterogeneity assessed at a single-cell resolution can potentially challenge narratives established using bulk-based characterization.

After deconstructing the ODE model results into enzyme-specific contributions with a multivariate PLSR analysis, we found that the top five most important enzymes in both the malignant and non-malignant models included NQO1, GLUD1, and G6PD, which counters the use of NQO1/CAT ratios for indicating β-lapachone potency in cells. The commonality of importance in both models indicated that both the drug-metabolizing enzyme itself and the enzymes that produce the main redox cofactor involved, NADPH, were crucial to determining impact of the drug on a cell’s redox state. Additionally, the higher importance of GLUD1 than NQO1 in the non-malignant model suggested that the production capacity of NADPH had a higher impact on the cell’s redox state than the expression of the actual enzyme metabolizing the drug. AQP3 appearing as a VIP enzyme in the malignant cell model was another interesting finding that suggests that if a cancerous cell can allow more H_2_O_2_ to leave the cell via AQP3, it will be able to maintain a better redox state. The rate of H_2_O_2_ export in malignant cells dictates bystander effects in which diffusion of the H_2_O_2_ can then go on to oxidatively stress neighboring cells. Encouragingly, a lower influence of AQP3 in the non-malignant cell simulations may reflect a lower potential for bystander effects to occur in healthy tissue even where drug quinone cycling occurs.

A current issue with scRNA-seq data is a large volume of dropouts, which leads to imputed values that are not true data [46]. Dropouts in the context of single-cell sequencing data refers to a lack of read counts for genes that may or may not be an accurate representation of gene expression. These occur due to the fact that single-cell sequencing is technically prone to not capturing every mRNA molecule within a cell while sequencing. Methods for both higher-quality sequencing and imputation are being developed, and as higher-quality datasets are published, this model can be updated to reflect that [47,48]. Additionally, the added value of spatial information from new spatial omics technologies could further improve the model. With the model currently representing a single-cell system, a multicellular model of all of the cells simultaneously with physical parameters included could better represent the tumor system and buildup and breakdown of ROS. Lastly, our model only predicts how these cells within patient samples would respond to β-lapachone. Working with directly validated samples is a more ideal workflow, and we look forward to testing these models’ accuracies if clinical data are made available in the future.

Altogether, this analysis demonstrates that developing a comprehensive enzymatic model of H_2_O_2_ generation and clearance using scRNA-seq data has the potential to identify the relative importance of various axes in the complex antioxidant network. Our modeling analysis points to highly heterogeneous intratumoral drug metabolism and patient-to-patient differences in how well β-lapachone may induce oxidative stress in malignant cells compared to non-malignant cells. We suggest that metrics other than NQO1:CAT should be considered when characterizing a HNSCC tumor and its capacity to respond to β-lapachone. These metrics include the expression of TXN, GPX1, POR, and NADPH-producing enzymes such as G6PD and GLUD1. Ultimately, the systems approach outlined here demonstrates the value of utilizing mechanistic modeling in conjunction with omics data to attain a more comprehensive understanding of the cellular redox state.

## Figures and Tables

**Table 1:** Additional rate terms for ODE model.

Reaction Name	Rate Term
β-lap permeation *	k_34_ * A^cells^ * ([β-lap^ext^]–[β-lapQ])
β-lap reduction	k_29_ * [β-lapQ] * [NADPH]
β-lap semioxidation	k_30_ * [β-lapHQ] * [O_2_]
β-lap oxidation	k_31_ * [β-lapSQ] * [O_2_]
superoxide dismutase	k_32_ * [O_2_^•−^]^2^
β-lap semireduction	k_33_ * [β-lapQ] * [NADPH]
β-lap semiquinone semireduction	k_33_ * [β-lapSQ] * [NADPH]
β-lap glutathionylation	k_35_ * [β-lapHQ] * [GSH]
Glutathionylated β-lap permeation *	k_36_ * A^cells^ * [β-lapHQ-SG]

* Permeation rate terms are divided by respective compartment volumes.

## Data Availability

Not applicable besides the use of already published data on the Gene Expression Omnibus (GSE103322).

## Supplementary Materials

The following supporting information can be downloaded at: https://www.mdpi.com/article/10.3390/antiox12030741/s1, Table S1: Model Parameters; Table S2: Model Initial Values.
